# Differential Toxicity of mDia Formin-Directed Functional Agonists and Antagonists in Developing Zebrafish

**DOI:** 10.3389/fphar.2018.00340

**Published:** 2018-04-10

**Authors:** Hunter LeCorgne, Andrew M. Tudosie, Kari Lavik, Robin Su, Kathryn N. Becker, Sara Moore, Yashna Walia, Alexander Wisner, Daniel Koehler, Arthur S. Alberts, Frederick E. Williams, Kathryn M. Eisenmann

**Affiliations:** ^1^Department of Cancer Biology, University of Toledo Health Science, Toledo, OH, United States; ^2^Department of Pharmacology and Experimental Therapeutics, University of Toledo Health Science, Toledo, OH, United States; ^3^Laboratory of Cell Structure and Signal Integration, Van Andel Research Institute, Grand Rapids, MI, United States

**Keywords:** SMIFH2, intramimics, formin, zebrafish, mDia, actin cytoskeleton, invasion, xenograft

## Abstract

The mammalian Diaphanous-related (mDia) formins are cytoskeletal regulators that assemble and, in some cases, bundle filamentous actin (F-actin), as well as stabilize microtubules. The development of small molecule antagonists and agonists that interrogate mDia formin function has allowed us to investigate the roles of formins in disease states. A small molecule inhibitor of FH2 domain (SMIFH2) inhibits mDia-dependent actin dynamics and abrogates tumor cell migration and cell division *in vitro* and *ex vivo* tissue explants. mDia formin activation with small molecule intramimics IMM01/02 and mDia2-DAD peptides inhibited glioblastoma motility and invasion *in vitro* and *ex vivo* rat brain slices. However, SMIFH2, IMMs, and mDia2 DAD efficacy *in vivo* remains largely unexplored and potential toxicity across a range of developmental phenotypes has not been thoroughly characterized. In this study, we performed an *in vivo* screen of early life-stage toxicity in *Danio rerio* zebrafish embryos 2 days post-fertilization (dpf) in response to SMIFH2, IMM01/02, and mDia2 DAD. SMIFH2 at concentrations ≥5–10 μM induced significant defects in developing zebrafish, including shorter body lengths, tail curvature and defective tail cellularity, craniofacial malformations, pericardial edema, absent and/or compromised vasculature function and flow, depressed heart rates and increased mortality. Conversely, IMM and mDia2 DAD peptides were minimally toxic at concentrations up to 10–20 and 50 μM, respectively. SMIFH2's therapeutic potential may therefore be limited by its substantial *in vivo* toxicity at functional concentrations. mDia formin agonism with IMMs and mDia2 DADs may therefore be a more effective and less toxic anti-invasive therapeutic approach.

## Introduction

Dynamic regulation of the cytoskeleton impacts cell motility in a variety of *in vitro* and *in vivo* tumor models. Formin family proteins regulate both actin and microtubule network dynamics. The mammalian Diaphanous (mDia) formins nucleate, elongate, and bundle filamentous actin (F-actin) (Castrillon and Wasserman, 1994; Romero et al., 2004; Kovar et al., 2006). mDia formins are essential to formation and function of protrusions underlying cell migration and invasion, including lamellipodia (Watanabe et al., 1997; Kurokawa and Matsuda, 2005; Eisenmann et al., 2007a; Gupton et al., 2007; Isogai et al., 2015b), filopodia (Peng et al., 2003; Yang et al., 2007; Mellor, 2010), invadopodia (Lizarraga et al., 2009), and plasma membrane blebs (Eisenmann et al., 2007a; Wyse et al., 2012). Independently, mDia formins stabilize microtubules (Gundersen and Bulinski, 1988; Tran et al., 2007; Bartolini et al., 2008; Thurston et al., 2012). Formin-regulated microtubules organize tracks for vesicle transport, underlie microtentacle formation, and facilitate pro-migratory Golgi re-orientation to aid in polarized cell movement (Nobes and Hall, 1999; Ridley et al., 2003; Yamana et al., 2006; Whipple et al., 2007; Andres-Delgado et al., 2012; Morris et al., 2014; Boggs et al., 2015; Bartolini et al., 2016). Drugs like taxol have made the cytoskeleton a clinically valid target. Thus, targeting formins with small molecules may be an effective therapeutic intervention with potential to impact both the microtubule and actin dynamics during cell division and migration.

mDia-formin-directed cytoskeleton regulation is mediated through the functional Formin Homology-2 (FH2) domain (Castrillon and Wasserman, 1994; Romero et al., 2004; Kovar et al., 2006). Auto-inhibited mDia formins maintain an inactive conformation when the C-terminal Dia auto-regulatory domain (DAD) is bound to the Dia inhibitory domain (DID), blocking FH2 domain functional activities (Watanabe et al., 1999; Alberts, 2001; Higgs, 2005; Li and Higgs, 2005). Rho-GTPase binding to the GTPase-binding domain (GBD) disrupts the DID-DAD binding to enable FH2 functional activities, including actin filament elongation and, in some cases, bundling.

Small molecule inhibitor of FH2 (SMIFH2) is a small molecule inhibitor that blocks functional formin activity in yeast and mammalian cells (Rizvi et al., 2009). This 2-thiooxodihydropyrimidine-4,6-dione derivative blocks mDia formin-mediated actin nucleation and elongation by disrupting F-actin binding. Only mDia1 and mDia2 FH2 inhibition was fully characterized biochemically *in vitro* for SMIFH2's effects upon rates of F-actin assembly and bundling (Rizvi et al., 2009). It remains uncertain if SMIFH2 inhibits other FH2-domain containing formin family members, as the FH2 domains of other non-mDia family formins (such as FMNL family members) were determined to share on average only ~25% sequence identity in multiple sequence alignments (Schonichen and Geyer, 2010). Nonetheless, SMIFH2 is considered by most to be a broad antagonist against FH2 domain-containing formins. SMIFH2 also disrupts microtubule networks, and alters Golgi orientation and cell polarity (Arden et al., 2015; Isogai et al., 2015a). Formin inhibition through dominant negative constructs, RNAi knockdown and small molecule antagonism has shown to be an effective anti-invasion strategy for halting cancer cell motility in some experimental systems (Poincloux et al., 2011) and could have clinical utility, based on some *in vitro* cancer cell models (Arden et al., 2015; Isogai et al., 2015a; Ziske et al., 2016). SMIFH2 alters cytoskeletal protrusion formation (Rizvi et al., 2009; Wyse et al., 2012; Borinskaya et al., 2015; Fattouh et al., 2015) with mixed effects on tumor cell migration (Rizvi et al., 2009; Pettee et al., 2014; Arden et al., 2015; Isogai et al., 2015a). SMIFH2 also disrupts normal fibroblast and epithelial cell cytoskeletal networks to promote aneuploidy (Rizvi et al., 2009; Efremov et al., 2015; Kim et al., 2015).

Studies show roles for formin antagonism impeding both the microtubule, as well as actin cytoskeletal dynamics. *In vitro*, low to moderate SMIFH2 concentrations are well-tolerated, but higher concentrations induce cytotoxic effects on mouse embryonic cells and NIH3T3 fibroblasts, HEK293T human embryonic kidney, MDA-MB-231 breast cancer, U2OS bone osteosarcoma, HCT116 colorectal carcinoma, U251 glioblastoma, and OVCA429 and ES2 ovarian carcinoma cells (Rizvi et al., 2009; Poincloux et al., 2011; Wyse et al., 2012, 2017; Pettee et al., 2014; Borinskaya et al., 2015; Fattouh et al., 2015; Isogai et al., 2015a; Ziske et al., 2016). This may be due to dual effective inhibition of both microtubule and actin dynamics within two-dimensional monolayers of cells. While 1 μM SMIFH2 disrupted ovarian cancer cell monolayer viability, higher concentrations were tolerated if cells were grown in three-dimensional (3D) spheroids (Ziske et al., 2016). This finding suggests that cells organized into physiologically-relevant organoid structures may confer increased tolerance to drug toxicity and formin antagonism. Evidence describing SMIFH2 efficacy and/or toxicity *in vivo*, however, are limited. A role for FMNL formins was shown in developing lumenized vasculature including intersegmental vessels (ISVs) in zebrafish (Hetheridge et al., 2012; Santos-Ledo et al., 2013), and SMIFH2 suppressed lumenized ISV development (Phng et al., 2015).

Formin agonists are less well studied. mDia2 agonist intramimics, or IMMs, are small molecule agonists that activate mDia2 through disruption of the autoregulatory interaction between the DAD and DID domains (Lash et al., 2013). We and others demonstrated that IMM01 and IMM02 not only enhance F-actin assembly, yet also stabilize microtubules to a lesser degree, and in some cases, cause cell cycle arrest and suppress xenografted tumor cells from generating tumors in adult mice (Lash et al., 2013; Arden et al., 2015). We also demonstrated in glioblastoma multiforme (GBM), IMM agonists, and their pegylated- (PEG) DAD domain peptide counterparts are superior to SMIFH2-mediated antagonism in blocking directional GBM cell migration and invasion *in vitro* in multi-cellular 3D spheroids, and in an *ex vivo* rat brain slice model of spheroid invasion (Arden et al., 2015). Interestingly, agonists did not induce cell death in GBM cell lines, and anti-invasive effects were reversible in washout experiments. Thus, it appears that the mechanism of actin in mDia formin agonists in cells is less toxic and directed toward halting invasion, as opposed to proliferation. A more comprehensive *in vivo* study of efficacy and/or toxicity of mDia agonists, in particular PEG-DAD, remains untested.

*Danio rerio* zebrafish are highly predictive for evaluating the toxicity of soluble compounds in mammals, due in part to significant gene conservation and organ homology between zebrafish and humans/mice. Soluble compounds can be placed directly into the embryo medium for passive uptake (Goldsmith, 2004; Shin et al., 2005; McGrath and Li, 2008; Goldsmith and Jobin, 2012; Salvaggio et al., 2016). In this study, toxicity of SMIFH2, IMMs and a newly developed PEG-DAD peptide were evaluated through zebrafish embryo early life stage toxicity screening. We found that while IMM and PEG-DAD compounds were well tolerated in zebrafish embryos, SMIFH2 induced significant toxicity to embryos at concentrations below those shown to be functionally antagonistic to F-actin dynamics *in vitro*. SMIFH2's therapeutic potential may therefore be limited by substantial *in vivo* toxicity at functional concentrations. Our data also point toward an emerging role for mDia2 agonists as well-tolerated therapeutic compounds with anti-invasive activities in several cancer types.

## Materials and methods

### Zebrafish housing, maintenance, and breeding

Adult *AB* and Tg *(fli1a:EGFP) Danio rerio* were from ZIRC (University of Oregon, Eugene, OR). This study was carried out in accordance with the recommendations in accordance with University of Toledo IACUC-approved regulations/protocols. Tg (*fli1a:EGFP*) fish express cytoplasmic EGFP within endothelial and endocardial cells, and the entire vascular system via the fli1a tissue-specific promoter (Lawson and Weinstein, 2002a,b). Adults were bred weekly in an Aquatic Habitats Mass Embryo Production System (MEPES) from Pentair (Minneapolis, MN). Embryos were maintained in standard embryo medium (1 L filtered R/O water with 0.6 g Instant Ocean Sea Salt) at 28°C.

### Zebrafish drug toxicity screen

At 48 h post-fertilization (hpf), embryos were enzymatically de-chorionated using 2 mg/mL pronase from Millipore (Burlington, MA). Healthy embryos were transferred 5/well into standard 24-well, clear, flat-bottom plates containing standard embryo medium. Time 0 images were collected at 2X on an EVOS-FL microscope (Olympus Plan N 2x/1.00 objective) immediately prior to treatment.

Working concentrations of 0.1–10 μM SMIFH2 from Tocris (Bristol, UK) were prepared in standard embryo medium. Vehicle control was prepared by diluting equivalent DMSO (Sigma) volumes for 10 μM SMIFH2 in standard embryo medium. Working concentrations of 5, 10, and 20 μM IMM01 and 1, 5, and 10 μM IMM02 from Thermofisher (Waltham, MA) were prepared by diluting 100 mM stock into standard embryo medium. Vehicle controls were prepared by diluting equivalent volume of DMSO from Sigma (St Louis, MO) for 20 μM IMM-01 in standard embryo medium. Working concentrations of 1, 10, and 50 μM of both PEG-DAD-M and PEG-DAD-A (DAD-M is the wild-type mDia2 DAD sequence with M1041, while DAD-A is M1041A substitution and is a negative control) (from New England Peptide, Gardner, MA) were prepared by diluting 10 mM stock into standard embryo medium. Vehicle controls were prepared by diluting equivalent volume of diH_2_O for 50 μM PEG-DADs in standard embryo medium. Embryo medium from each well was replaced with 900 μl of treated embryo medium. Drug concentrations were maintained by exchanging medium every 24 h. Treatments were in triplicate with three wells prepared for each drug condition and five embryos/well. All screens were repeated at least three independent times.

### Embryo assessment and phenotype scoring

After 4–144 h post-treatment (hpt), brightfield or fluorescent images for each embryo were acquired using an EVOS microscope with a 2X objective. Tricaine anesthesia was used to orient the fish in a sagittal plane in order to obtain images clearly characterizing any defects (i.e., tail curvature/degradation, edema, and head shape), and does not interfere with heart rate phenotypes (Craig et al., 2006).

Treatment mortality was based on number of surviving embryos with a visible heartbeat at each time point. Embryo heart rates in beats per minute (bpm) were determined by manually counting the embryo heart beat for 10 s and multiplying that value by 6.

Early life stage toxicity defects were determined by manually examining blinded embryo images. These images were scored for tail curvature and degradation, pericardial edema, craniofacial characteristics, and development of sub-intestinal vessels (SIVs). Metamorph software measured zebrafish length from head to tail. For tail curvature, deviation observed above or below the straight edge was scored as tail curvature. Craniofacial defects (head shape) were blindly scored qualitatively as round, intermediate or square shapes with consideration of the embryo's orientation angle. SIVs are characterized as a loop of vessels protruding down into the posterior side of the embryonic sac. Fluorescence images at 21 hpt were evaluated and SIVs were scored as either present or absent.

### Statistical analysis

Graphical representations were prepared using GraphPad Prism software. One way ANOVA analysis was used to determine statistical significance, where *p* < 0.05 was considered statistically significant. Error bars represent standard errors (SE).

## Results

### SMIFH2 induces tail curvature and peripheral cell death in tails of developing zebrafish embryos

**Figure 1:**
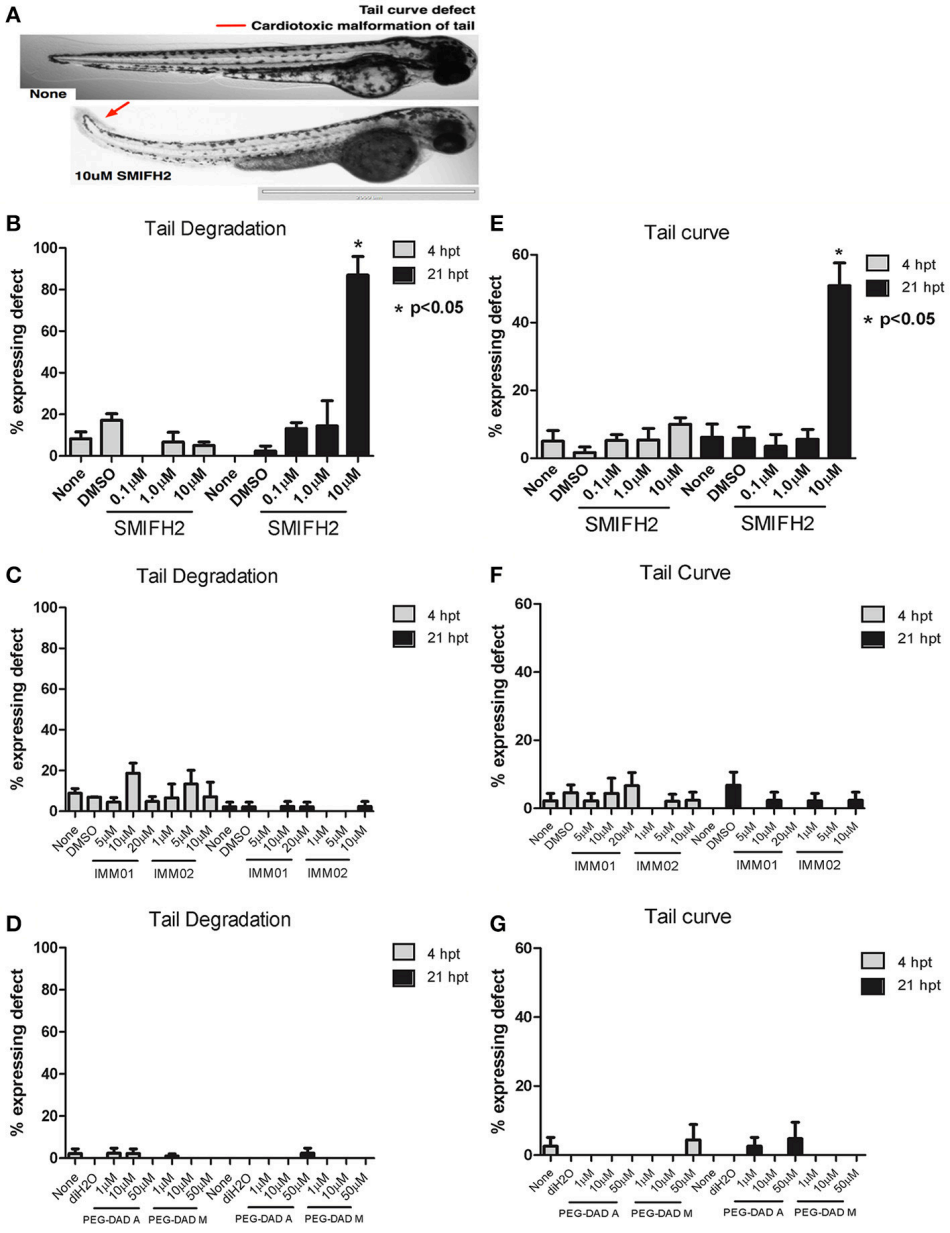
Formin antagonism induces tail curving and peripheral tail cell death in developing zebrafish embryos. **(A)** Representative 2x images at 21 hpt untreated (None) and in 10 μM SMIFH2 (arrow indicates peripheral tail cell death). Scale bar = 2,000 μm. **(B–D)** Tail degradation was scored for embryos in each untreated, vehicle, and SMIFH2 **(B)**, IMM **(C)**, and PEG-DAD **(D)** drug conditions. **(E-G)**. Tail curvature was scored for embryos in each untreated, vehicle, and SMIFH2 **(E)**, IMM **(F)**, and PEG-DAD **(G)** drug condition. The screens were performed in triplicate, five embryos/well and the experiment was repeated three times. *p*-Value is relative to untreated condition. Error bars indicate SE.

We performed toxicity screens on 2 dpf zebrafish embryos, testing 0.1–10 μM DMSO (vehicle), SMIFH2, and 1–10 μM IMMs (or DMSO) or 1–50 μM PEG-DADs (or diH_2_0) for up to 28 hpt. We first evaluated tail curvature and tail morphologies in treated zebrafish embryos (Figures 1A–G). The untreated, DMSO-, and diH_2_O-treated zebrafish had straight tails at 21 hpt, as did embryos treated with all concentrations of IMM01, IMM02, and PEG-DADs, including the wild-type DAD sequence, PEG-DAD M, as well as the alanine-substituted negative control, PEG-DAD A (Figures 1F,G). Embryos treated with 0.1 and 1 μM SMIFH2 presented with straight tails (Figures 1A,E). However, 10 μM SMIFH2 induced dramatic and statistically significant upwards tail curvature 21 hpt (>5-fold increase relative to controls), and curvatures appeared to begin posterior of the end of the yolk extension (Figures 1A,E). Furthermore, the upwardly curved tails from 10 μM SMIFH2-treated embryos also showed highly disorganized peripheral tail cellular structure relative to controls (Figures 1B–D), accompanied by tail tip malformation with discoloration and deterioration, indicative of peripheral tail cell death. At >10 μM SMIFH2, cells rapidly sloughed off tails within 21 hpt (not shown). This may be indicative of a lack of perfusion caused by overall cardiotoxicity of SMIFH2 and mDia functional suppression. In contrast, IMM and PEG-DAD mDia2 agonist-treated embryos did not have compromised tail structures, relative to untreated and vehicle treated controls.

### Antagonism induces craniofacial malformations in developing zebrafish embryos

**Figure 2:**
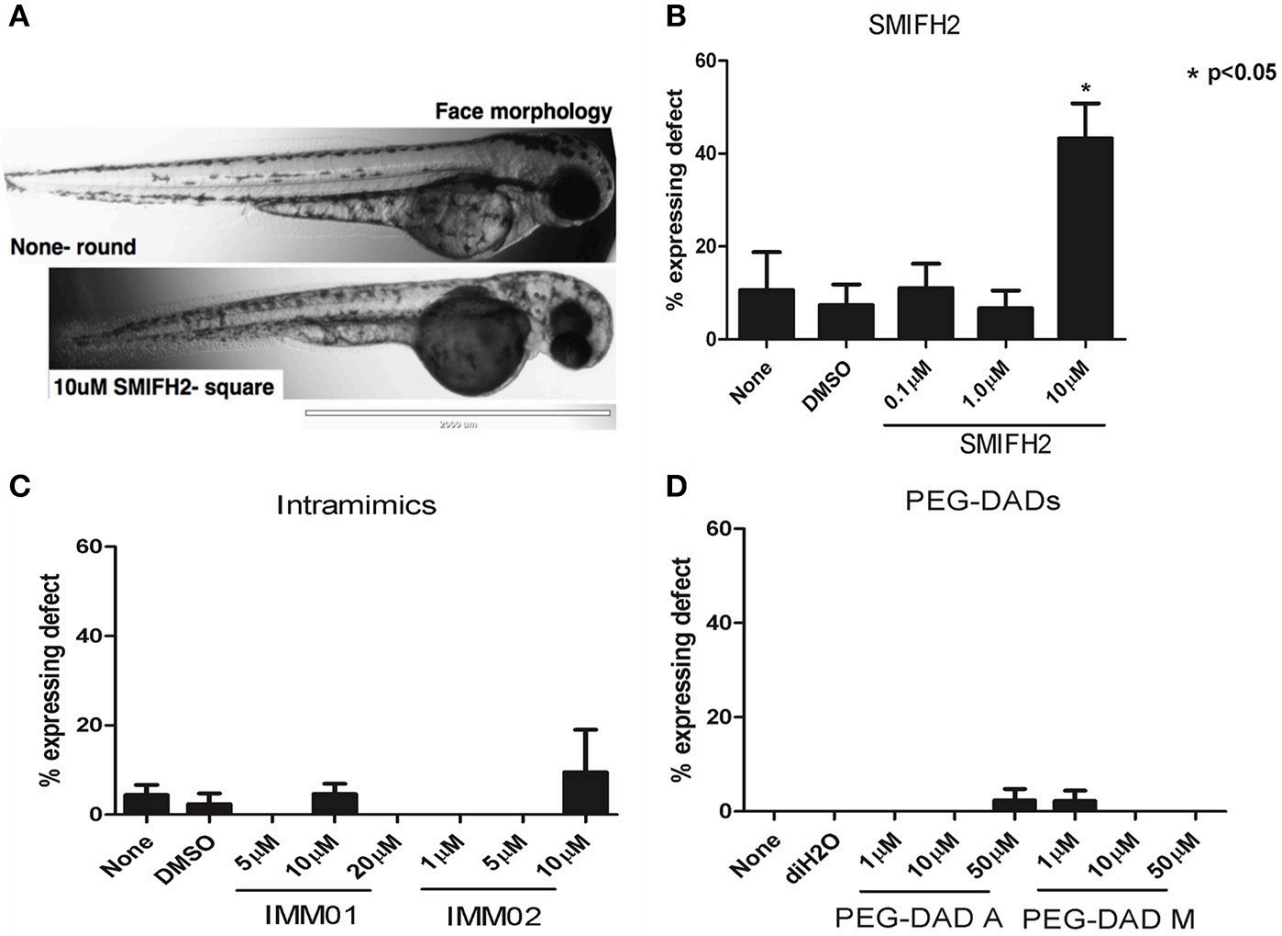
Formin antagonism induces craniofacial defects in developing zebrafish embryos. **(A)** Representative 2x images at 21 hpt untreated (None) and in 10 μM SMIFH2. **(B–D)** Craniofacial defects were scored for embryos in each untreated, vehicle, and SMIFH2 **(B)**, IMM **(C)**, and PEG-DAD **(D)** drug conditions. The screens were performed in triplicate, five embryos/well and the experiment was repeated three times. Values are represented as % expressing squared faces per condition. *p*- value is relative to untreated condition. Error bars indicate SE. Scale bars = 2,000 μm.

We next assessed craniofacial mutations observed in treated zebrafish embryos. Untreated, DMSO-, and diH_2_O-treated embryos had characteristic rounded facial structures, elongated snouts, with two distinct eyes (Figure 2A, upper panel), as did embryos treated with all concentrations of IMM01, IMM02, PEG-DAD-M, and PEG-DAD-A (Figures 2A,C,D). Craniofacial features in embryos treated with 0.1 and 1 μM SMIFH2 were unaffected, while 10 μM SMIFH2-treated embryos had a statistically significant increase in blunted, shortened, or squared-off facial morphologies (5-fold increase relative to controls; Figures 2A,B), indicative of a developing jaw defect and perhaps due to deficiencies in local vascularization or circulation impacting jaw growth (Teraoka et al., 2002). The eyes were also misplaced, fused, or near-fused in SMIFH2-treated embryos having the squared craniofacial morphology.

### Antagonism induces pericardial edema in developing zebrafish embryos

**Figure 3:**
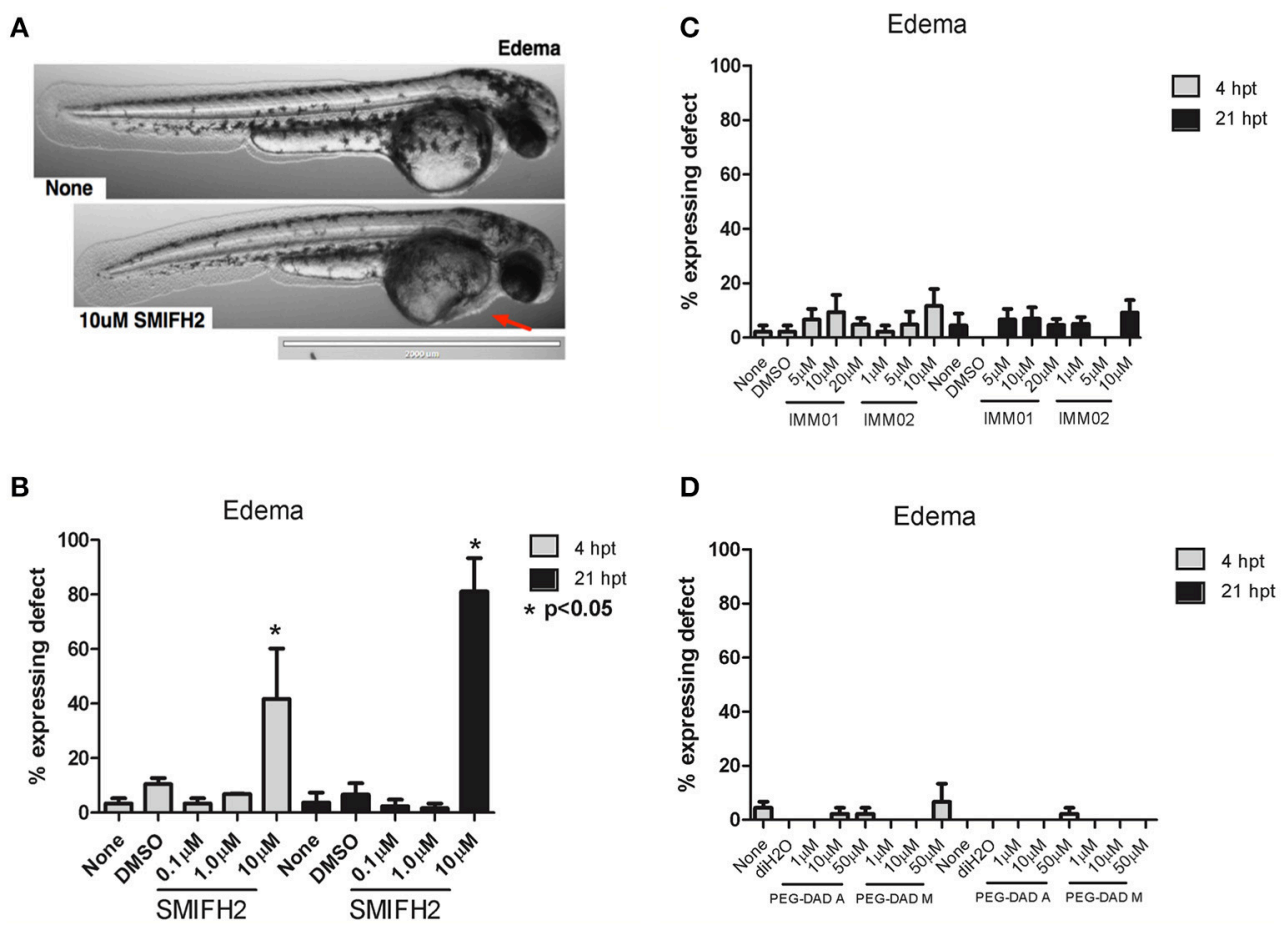
Formin antagonism induces pericardial edema in developing zebrafish embryos**. (A)** Representative 2x images at 21 hpt without treatment (None) and in 10 μM SMIFH2 (arrow indicates edema). Scale bar = 2,000 μm. **(B–D)** Pericardial edema was scored for embryos in each untreated, vehicle, and SMIFH2 **(B)**, IMM **(C)**, and PEG-DAD **(D)** drug conditions. The screens were performed in triplicate, five embryos/well and the experiment was repeated three times. *p*-value is relative to untreated condition. Error bars indicate SE.

We next quantified pericardial edema in treated embryos. In control, DMSO-, and diH_2_O-treated embryos there was no evidence of pericardial edema (Figure 3). Similarly, there was no edema in those embryos treated with increasing concentrations of IMM01, IMM02, PEG-DAD-M, and PEG-DAD-A, relative to controls (Figures 3C,D). Embryos treated with 0.1 and 1 μM SMIFH2 were also normal with respect to pericardial edema; However, in 10 μM SMIFH2-treated embryos, we observed clear masses anterior to the heart yet posterior to the head (Figure 3A). Nearly 90% of 10 μM SMIFH2-treated embryos had pericardial edema, relative to 10% of embryos treated with respective vehicle or mDia2 agonists (Figure 3B). Pericardial edema has previously been attributed to increases in vascular permeability of the proximal vessels, or impaired development of the heart itself (Hill et al., 2004).

### Antagonism impairs vascular flow in developing zebrafish embryos

We next wished to investigate the dynamics of vascular flow within treated embryos upon mDia antagonism. Hence, we treated developing zebrafish embryos with DMSO, or 0.1–10 μM SMIFH2 for 4 hpt. Live imaging of the cardinal vein anterior to the urogenital opening of tricaine-treated embryos was then performed to assess vascular flow posterior to the yolk extension. The vascular flow rates of 0.1 and 1 μM SMIFH2-treated embryos resembled untreated or DMSO-treated embryos (Movies S1–S4, respectively). A striking difference was seen in 5 and 10 μM SMIFH2-treated embryos, where vascular flow was suppressed and nearly halted, respectively (Movies S5, S6).

### Antagonism impacts heart rates in developing zebrafish embryos

**Figure 4:**
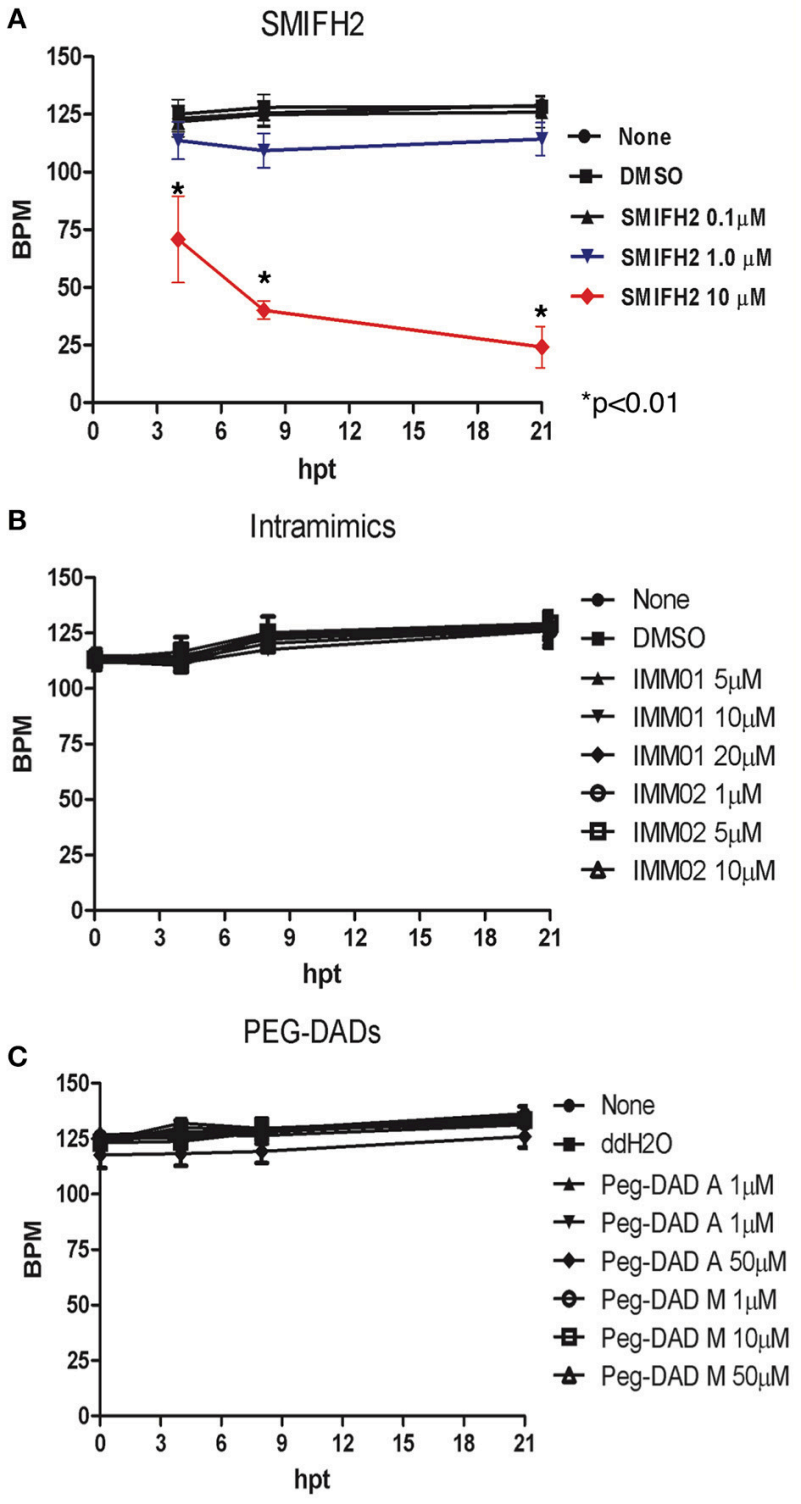
Formin antagonism induces heart rate depression in developing zebrafish embryos. Heart rates were scored in: untreated, vehicle, SMIFH2 **(A)**, IMM01 and IMM02 **(B)**, and PEG-DAD-A and PEG-DAD-M **(C)**. The screens were performed in triplicate, five embryos/well and the experiment was repeated three times. Error bars represent SE.

Finally, we assessed heart rates in developing embryos treated with IMM01, IMM02, PEG-DADs, and SMIFH2 through 21 hpt. Heart rates for untreated, DMSO-, diH_2_O-, IMM01-, IMM02-, PEG-DAD-A-, and PEG-DAD-M- and 0.1 μM SMIFH2-treated embryos remained near 120–130 bpm, with little variation across 21 hpt (Figure 4). Exposure to 1 μM SMIFH2 suppressed bpm about 10% relative to controls, and this was nearly recovered to control levels within 21 hpt (Figure 4A). Embryos treated with 10 μM SMIFH2 revealed a rapid heart rate decline as early as 4 hpt. We observed a rapid decline in survival by 21 hpt in these embryos.

## Discussion

As major regulators of cellular functions including cytokinesis, vesicular trafficking, and cell motility, mDia formins were proposed as therapeutic targets in a wide-variety of pathologies, including invasive and metastatic cancers. There are at least two approaches to manipulating formin activity: antagonism and agonism. Both have been reported to target both the actin and microtubule cytoskeletons to varying degrees in different two-dimensional cellular models. An antagonist, the small molecule inhibitor SMIFH2, is a widely-used tool used to study mDia function *in vitro* in a variety of human cells. This study reveals cytotoxicity *in vivo* in developing zebrafish at ≥5–10 μM SMIFH2. Alternatively, mDia2 intramimics, IMM01 and IMM02, as well as PEG-DADs induced no significant cytotoxicity. We documented defects in embryo lengths, embryo tail curves and cellularity, pericardial edema, craniofacial development, vascular development, and heart rates. Ultimately zebrafish embryos treated with ≥10 μM SMIFH2 were no longer viable.

Although this study was performed in developing zebrafish embryos, embryo toxicity screens are highly predictive of mammalian drug toxicity (Langheinrich, 2003; Zhang et al., 2003; Kari et al., 2007). Zebrafish are anatomically, physiologically and molecularly similar to mammals and most major organs, including nervous system, cardiovascular system, intestines, liver, and kidneys are established by 5 dpf (McGrath and Li, 2008). The circulatory system of zebrafish is a common site of drug toxicity. Zebrafish embryos are ideal for assessing cardiotoxicity as the heart is formed and functional by 26 hpf (Baker et al., 1997; Chakravarthy et al., 2014), and all major vessels are present with active circulation by 28 hpf (Kari et al., 2007). At 28 hpf, angiogenesis is underway within the anterior somites, consistent with tail curvature and cellular defects in SMIFH2-treated embryos (Figure 2). Zebrafish embryos survive for up to a week without intact circulation (Stainier, 2001; Kari et al., 2007), supporting our findings that embryos persist despite lack of blood circulation while developing pericardial edema and cranial-facial abnormalities.

SMIFH2's toxicity at ≥5–10 μM in developing zebrafish embryos may caution against its therapeutic utility in mDia formin antagonism. SMIFH2 concentrations of ≥10 μM predominate *in vitro* tumor cell invasion studies (Arden et al., 2015; Isogai et al., 2015a; Ziske et al., 2016; Wyse et al., 2017). However, purified biochemical assays (Rizvi et al., 2009) indicated that 4 μM SMIFH2 blocked mDia formin-dependent actin nucleation and elongation, whereas 2.5 μM induced actin cable de-polymerization. Thus, at lower concentrations, SMIFH2 is effective- at least in a closed, purified system. *In vitro* in normal cells, 2.5 μM SMIFH2 disrupted cytokinesis in NIH3T3 fibroblasts and 5 μM SMIFH2 dramatically decreased MEF spreading (Iskratsch et al., 2013). SMIFH2 (0.1–1 μM) also enhanced Taxol or Cisplatin anti-proliferative effects in ovarian cancer spheroid cultures (Ziske et al., 2016). Here, no significant early-life stage embryo toxicity was detected at 0.1 and 1 μM SMIFH2, but 5 μM caused substantial cardiotoxic defects in embryos (data not shown) and impaired vascular flow (Movie S5) through 21 and 4 hpt, respectively. Recently it was suggested that 5 μM SMIFH2 treatment for 15 h starting at 31 hpf in zebrafish embryos suppressed F-actin stability at endothelial junctions, as serrated F-actin cables were increased (an indicator of F-actin remodeling) (Phng et al., 2015). Further studies are warranted to definitively assess the functional efficacy of SMIFH2-directed mDia formin inhibition at ≤5 μM both *in vitro* and *in vivo* and to ascertain if both the microtubule and actin filament systems are being impacted. Cell-type specificity in toxicity may also exist *in vitro* and *in vivo*, as we previously showed 10 and 20 μM SMIFH2 minimally impacted ES-2 and SKOV3 ovarian cancer 3D multi-cellular spheroid viability (Ziske et al., 2016), while 40 μM SMIFH2 was not acutely toxic in treated adult rat brain slices cultured up to 48 h (Arden et al., 2015). SMIFH2 toxicity studies in different zebrafish developmental stages (i.e., juvenile, adult) are also currently in progress.

Our results support previous studies conducted in mouse models suggesting that formin antagonism can have a significant effect on homeostatic systems. *DRF1* (encoding mDia1 protein) knockout mice exhibited disrupted myelopoiesis and erythropoiesis resembling the pre-leukemic human diseases chronic myeloproliferative syndrome (MPS) and myelodysplastic syndrome (MDS) (Peng et al., 2007). mDia1 knockout mice also showed signs of dehydration, anemia, and hepatic dysplasia (Peng et al., 2007), and had reduced numbers of T lymphocytes in the bone marrow, spleen, thymus, and blood (Eisenmann et al., 2007b). Furthermore, *DRF2* (encoding mDia3 protein) suppression was correlated with primary ovarian failure (Wynshaw-Boris et al., 1997; Bione et al., 1998; Tominaga et al., 2002), while in *DRF3* (encoding mDia2 protein) knockdown cells and mice, severe defects in erythrocyte maturation and enucleation were seen (Ji et al., 2008; Watanabe et al., 2013; Mei et al., 2016). These data suggest a critical role for mDia formins during both hematopoiesis and hemostasis, thus indicating caution in inhibiting global or, in some cases, tissue-specific mDia expression and/or functional antagonism *in vivo* in clinical applications.

In contrast, formin activation and not functional suppression may be more appropriate clinical strategy to target tumor cell motility, and potentially proliferation. Whereas, mDia2 suppression drove alternative modes of tumor cell motility in cervical, ovarian, breast, and prostate cancers, and hepatocarcinoma models (Eisenmann et al., 2007a; Di Vizio et al., 2009; Hager et al., 2012; Wyse et al., 2012; Pettee et al., 2014), glioblastoma cells *in vitro* and *ex vivo* spheroid-rat brain slice models showed IMM-induced mDia2 agonism more effectively blocked glioblastoma migration and invasion than antagonism, with no toxicity in rat brain slice explants (Arden et al., 2015). IMM compounds decreased hepatocarcinoma tumor burden *in vivo* in mice with limited side effects (Lash et al., 2013). Thus, endogenous mDia formin activation may ultimately prove a more viable therapeutic avenue to targeting tumor cell motility in future studies.

## Author contributions

HL, AT, KL, RS, KB, SM, YW, AW, and DK: performed the experiments; HL, AT, and KL: drafted the manuscript; KE, AA, and FW: conceived of the experiments; KE and FW: provided funding sources and edited the paper; AA: provided invaluable reagents. All authors read and approved the submitted version (except AA, who passed away prior to submission).

### Conflict of interest statement

The authors declare that the research was conducted in the absence of any commercial or financial relationships that could be construed as a potential conflict of interest.
