# Supplementary material for: Differential Toxicity of mDia Formin-Directed Functional Agonists and Antagonists in Developing Zebrafish
Source: Front Pharmacol. 2018 Apr 10;9:340. doi: 10.3389/fphar.2018.00340 (PMC5902741; doi:10.3389/fphar.2018.00340)
Supplement: Supplementary file 7 [file DataSheet1.DOCX]

**SUPPLEMENTAL MOVIE LEGENDS**

**Movie 1: Vascular flow in untreated embryos.** Untreated 48 hpf embryos live imaged for 10s. Shown is the cardinal vein anterior to the yolk sac extension, and proximal to the urogenital opening. Scale bar = 100μm.

**Movie 2: Vascular flow in DMSO-treated embryos.** 48 hpf embryos were treated for 4h with DMSO and live imaged for 10s. Shown is the cardinal vein anterior to the yolk sac extension, and proximal to the urogenital opening. Scale bar = 100μm.

**Movie 3: Vascular flow in 0.1μM SMIFH2-treated embryos.** 48 hpf embryos were treated for 4h with SMIFH2 and live imaged for 10s. Shown is the cardinal vein anterior to the yolk sac extension, and proximal to the urogenital opening. Scale bar = 100μm.

**Movie 4: Vascular flow in 1μM SMIFH2-treated embryos.** 48 hpf embryos were treated for 4h with SMIFH2 and live imaged for 10s. Shown is the cardinal vein anterior to the yolk sac extension, and proximal to the urogenital opening. Scale bar = 100μm.

**Movie 5: Vascular flow in 5μM SMIFH2-treated embryos.** 48 hpf embryos were treated for 4h with SMIFH2 and live imaged for 10s. Shown is the cardinal vein anterior to the yolk sac extension, and proximal to the urogenital opening. Scale bar = 100μm.

**Movie 6: Vascular flow in 10μM SMIFH2-treated embryos.** 48 hpf embryos were treated for 4h with SMIFH2 and live imaged for 10s. Shown is the cardinal vein anterior to the yolk sac extension, and proximal to the urogenital opening. Scale bar = 100μm
